# Role of the Contralesional Hemisphere in Post-Stroke Recovery of Upper Extremity Motor Function

**DOI:** 10.3389/fneur.2015.00214

**Published:** 2015-10-16

**Authors:** Cathrin M. Buetefisch

**Affiliations:** ^1^Emory University, Atlanta, GA, USA; ^2^Georgia Institute of Technology, Atlanta, GA, USA

**Keywords:** transcranial magnetic stimulation, motor cortex reorganization, neurorehabilitation of motor function, motor stroke recovery, functional magnetic resonance image

## Abstract

Identification of optimal treatment strategies to improve recovery is limited by the incomplete understanding of the neurobiological principles of recovery. Motor cortex (M1) reorganization of the lesioned hemisphere (ipsilesional M1) plays a major role in post-stroke motor recovery and is a primary target for rehabilitation therapy. Reorganization of M1 in the hemisphere contralateral to the stroke (contralesional M1) may, however, serve as an additional source of cortical reorganization and related recovery. The extent and outcome of such reorganization depends on many factors, including lesion size and time since stroke. In the chronic phase post-stroke, contralesional M1 seems to interfere with motor function of the paretic limb in a subset of patients, possibly through abnormally increased inhibition of lesioned M1 by the contralesional M1. In such patients, decreasing contralesional M1 excitability by cortical stimulation results in improved performance of the paretic limb. However, emerging evidence suggests a potentially supportive role of contralesional M1. After infarction of M1 or its corticospinal projections, there is abnormally increased excitatory neural activity and activation in contralesional M1 that correlates with favorable motor recovery. Decreasing contralesional M1 excitability in these patients may result in deterioration of paretic limb performance. In animal stroke models, reorganizational changes in contralesional M1 depend on the lesion size and rehabilitation treatment and include long-term changes in neurotransmitter systems, dendritic growth, and synapse formation. While there is, therefore, some evidence that activity in contralesional M1 will impact the extent of motor function of the paretic limb in the subacute and chronic phase post-stroke and may serve as a new target for rehabilitation treatment strategies, the precise factors that specifically influence its role in the recovery process remain to be defined.

## Introduction

With the introduction of relatively sophisticated neuroimaging techniques, such as positron emission tomography (PET) and functional and structural magnetic resonance imaging (MRI), and novel electrophysiological techniques, such as transcranial magnetic stimulation (TMS), studying the underlying mechanisms of motor recovery after stroke in humans have become increasingly feasible. In 1991, Chollet et al. (1) reported for the first time the activation of bilateral sensorimotor cortices in stroke patients moving their affected hand and suggested that ipsilateral motor projection may play a role in recovery. This claim was further substantiated in 1993 by Carr et al. (2) who used TMS of the primary motor cortex (M1) to probe the functional integrity of the corticospinal tract (CST) after stroke. He reported that, in patients with poor motor outcome, TMS applied to the motor cortex of the hemisphere affected by stroke (ipsilesional M1) did not produce detectable motor-evoked potentials (MEPs), indicating disrupted function of the CST. However, when TMS was applied to the motor cortex of the hemisphere spared by the stroke (contralesional M1), MEPs were detected in both the hands. These findings suggested abnormal corticospinal projections from the contralesional M1 to muscles of the affected hand (see below for more detailed discussion).

In the following years, the role of the contralesional M1 in motor recovery after stroke and its potential as new target for rehabilitation efforts have been a topic of intense research efforts in humans and animal stroke models (3–5). As this field moved forward, it became apparent that several factors may impact the role of contralesional M1 in the control of the paretic hand movements and that even in healthy intact brain the ipsilateral M1 (corresponding to the contralesional M1 in paretic hand movements) is active in the control of strictly unilateral hand movement (6–11). In the context of the incomplete understanding of the ipsilateral M1 in motor control, the interpretation of findings pertaining to the role of contralesional M1 (corresponding to the ipsilateral M1 in intact human) in motor recovery after stroke remains problematic.

In this review, the evidence for contralesional M1 activity in recovery of hand function after stroke will be discussed. In the first part of this review, I will summarize the advances in our understanding of motor control of hand movements as they pertain to a better understanding of contralesional M1 function in motor recovery of hand movements. There is emerging evidence that ipsilateral M1 (corresponding to contralesional M1 in stroke patients) is active even in healthy subjects, depending on age and motor task demands (11–14). Motor task-dependent activity of ipsilateral M1 and the interaction between M1s may contribute to the contradicting data in contralesional M1 in stroke patients, where stroke-related motor impairment impacts the demand of a given motor task. In the second part of the review, I will discuss data available from animal stroke models and humans after stroke pertaining to the role of contralesional M1 reorganization in post-stroke recovery. Finally, I will discuss in which way neurorehabilitation science can leverage on the knowledge of contralesional M1 reorganization to develop new and effective rehabilitation treatment strategies.

## Ipsilateral M1 and Interhemispheric Interaction in the Control of Hand Movements in Intact Man

### The contribution of ipsilateral M1 and its corticospinal connections in the control of hand movements

**Figure 1:**
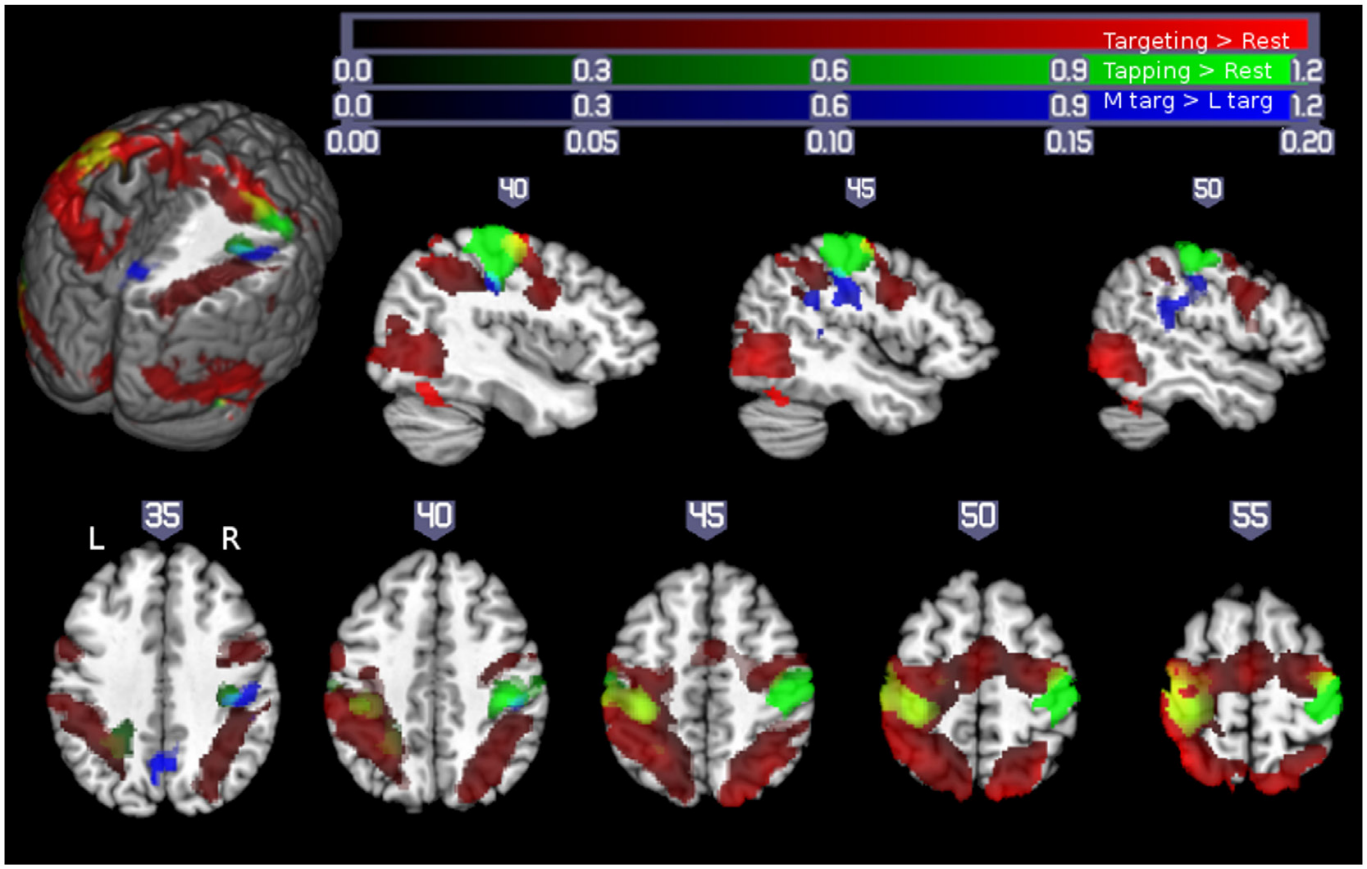
**Motor demand-dependent activation of motor cortices using a pointing task: pointing and finger tapping tasks related brain activation: Activity related to the pointing task (collapsed across XL, L, and M targets) is indicated in red**. Activation related to right- and left-handed finger tapping is indicated in green, with overlap between finger tapping and pointing task performance shown in yellow. Note that while there was extensive bilateral activation for the pointing task, M1 activation in the finger tapping tasks was only seen contralateral to the performing hand, so that the left hemisphere is solely due to right-handed finger tapping (with left hemisphere yellow areas show overlap between right-handed finger tapping and right-handed pointing task performance) and the right hemisphere activity is solely due to left-handed finger tapping (yellow colors in the right hemisphere show overlap between activity due to the right-handed targeting task and left-handed finger tapping task, outlined with a yellow border for ease of visualization). Significant activation related to increasing motor demand (M targets > L targets) is indicated in blue (overlap between this region and left-handed finger tapping shown in cyan, outlined for clarity). All activations are shown overlaid on the Colin27 template in standard space, thresholded at a corrected *p* < 0.05 (uncorrected threshold *p* < 0.005 and cluster size >2360 mm^3^). Increased color intensity corresponds to higher estimates of percent signal change. Cuts in the three-dimensional rendering are shown at *x* = 0, *y* = −15, and *z* = 35. The right hemisphere is depicted in the upper panel. The right (R) and left (L) side of the brain are indicated in the lower panel. Numerical labels above each slice show slice coordinates in the *x* dimension (sagittal sections) or *z* dimension (axial sections) (11).

In fMRI studies of unilateral hand motor performance in intact man, strictly contralateral M1 activation was demonstrated by some investigators (15, 16) while bilateral M1 activation was observed by others (6, 11, 17–19). Increased ipsilateral M1 was demonstrated in tasks with higher accuracy or complexity demands (6–8, 11, 17, 20). However, the interpretation of these neuroimaging data was limited by measuring qualitatively different movements where the tasks were not being matched for their kinematics (e.g., force, amplitude, and frequency) and by lacking the verification of a strictly unilateral execution of the motor task during the acquisition of imaging data. Measuring unilateral performance is important as without it, the presence of bilateral upper extremity activity with increasing difficulty of the task referred to as “mirror movements” cannot be ruled out and may contribute to observed bilateral M1 activation. In our recent study of healthy middle-aged people (*n* = 13, 10 females, age 55.4 ± 10.9 years), subjects performed a pointing task with a joy stick. By decreasing the size of the target, the demand on accuracy was parametrically increased while participating muscle groups and movement kinematic were kept the same. Unilateral performance was verified with electromyographic (EMG) recording from upper extremity muscles. As illustrated in Figure 1, performance of the pointing task (collapsed across different target sizes) resulted in extensive activation of bilateral sensorimotor cortex in the precentral and postcentral gyri/sulci (Figure 1, red). This contrasts with activation arising from the qualitatively different finger tapping task (Figure 1, green/yellow), which resulted in activation restricted to contralateral sensorimotor areas and the corresponding ipsilateral cerebellum. Of note is that ipsilateral M1 activation in the pointing task is largely anterior to the activation arising from the tapping task executed by the contralateral hand.

While there is evidence for ipsilateral corticospinal projections in humans, evidence for the control of the hand movements via ipsilateral corticospinal connections is weak. In intact humans, stimulation of M1 using TMS elicits MEPs in ipsilateral hand muscles but these are difficult to obtain and require high stimulation intensity and pre-innervations of the target muscle (21). In non-human primates, recording of ipsilateral M1 neurons during upper limb movements demonstrate that cells in iM1 are modulated by the task but that the timing of this activity is best correlated with weak muscle activity in the contralateral non-moving arm (22). Alternatively, task-related effects in the ipsilateral M1 could be mediated by corticoreticulospinal connections. In contrast to corticospinal connection, corticoreticulospinal projections are bilateral and are thought to be involved in the execution of selective finger movements (23). The involvement of this pathway is supported by TMS-derived evidence of longer latencies of MEPs elicited in the ipsilateral hand muscles (21). One could also argue that this M1 area may be concerned with the integration of afferent input from other motor areas. Recent evidence of bilateral M1 projections from posterior parietal (24, 25) and dorsal premotor areas, likely conveying some task-related information such as visuospatial and motor planning information, support a more indirect effect and the notion that M1 functions at a higher level in motor control by integrating afferent information and then generating a descending motor command that defines the spatiotemporal form of the movement (26). A higher level role for M1 in motor control is also supported by the results of a recent repetitive TMS (rTMS) study where low-frequency rTMS applied to left M1 improved performance in both hands for the task with the highest demand on precision while performance remained unchanged for the tasks with lower demands (14).

### Interhemispheric interaction in the control of hand movements in intact humans

In addition to the corticospinal projections and ipsilateral corticocortico connections, motor areas of the two hemispheres are interconnected to each other and interact in the execution of motor tasks. Improved performance after transiently inhibiting the ipsilateral M1 by means of low-frequency rTMS (14, 27, 28) could indicate that there may be a need for suppression of task performance related ipsilateral M1 excitatory activity. Because the relationship between the two primary motor cortices is impacted by stroke (4, 5, 29) and topic of great interest in neuromodulation treatment approaches targeting the contralesional M1 (3), this topic will be reviewed for the intact brain.

**Figure 2:**
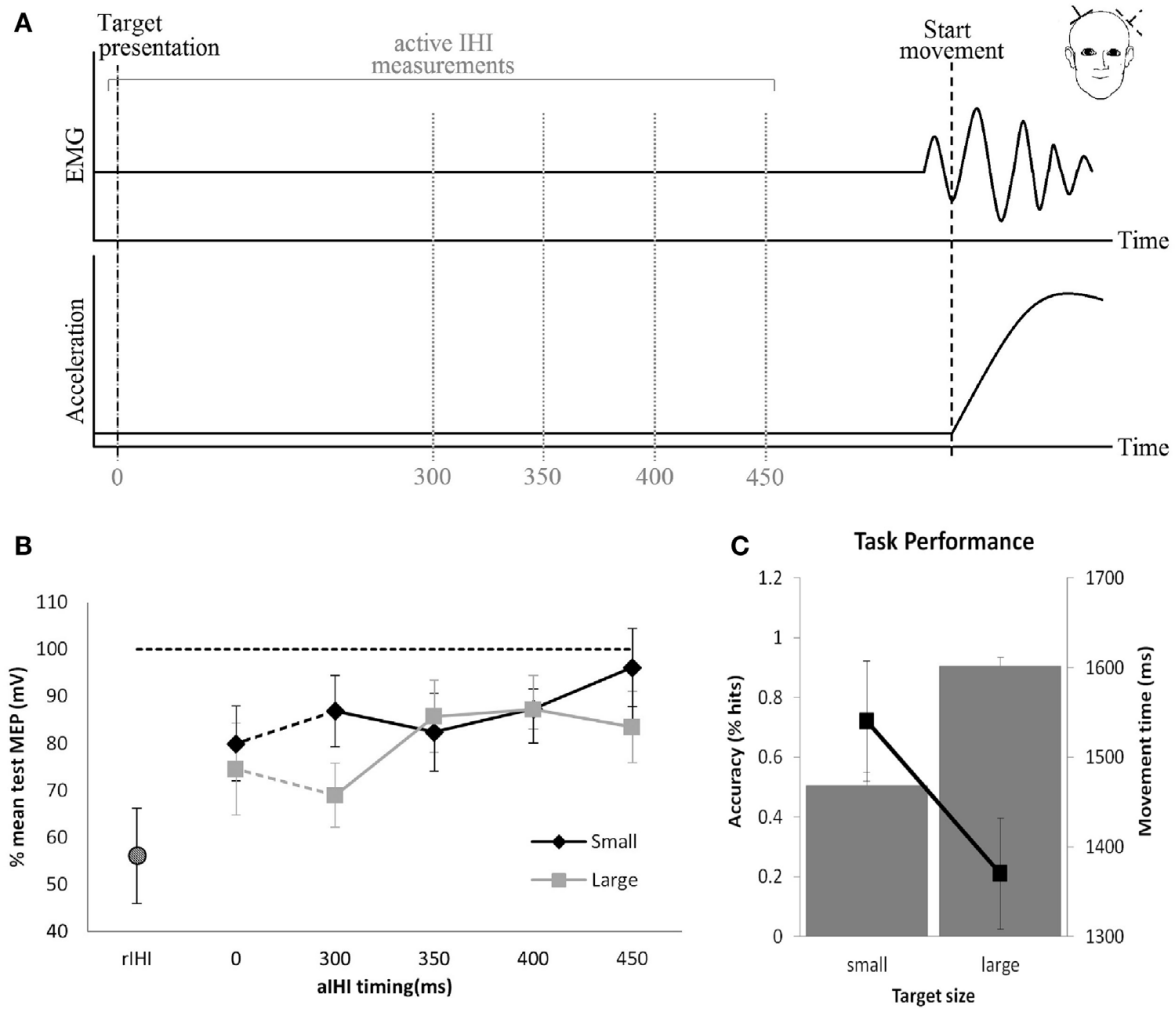
**Resting and active interhemispheric inhibition (IHI): (A)** IHI can be demonstrated by applying a conditioning stimulus to M1, which inhibits the size of the motor-evoked potential (MEP) produced by the test stimulus applied to the homotopic area of the opposite M1. These measures are obtained during rest (resting IHI, rIHI) or in the pre-movement period during preparation of a movement (active IHI). **(B)** During rest, there is significant rIHI (round symbol) from one M1 on the other M1. Active IHI (rectangular symbol) decreases immediately prior to the movement onset depending on kinematics of the movement **(B,C)**. **(B,C)** Pointing to a large target with less demand on accuracy (square) results in less reduction of active IHI compared to pointing at a small target (diamond) with high demand on accuracy (33).

The main structure connecting the motor areas is the corpus callosum. Connections between primary motor areas are less abundant than premotor areas and primarily excitatory [for detailed review, see Ref. (5)]. Interhemispheric inhibition (IHI) can be demonstrated with TMS by applying a conditioning stimulus (CS) to one M1 and a test stimulus (TS) to the homotopic area of the other M1 (30) (Figure 2). The CS inhibits the size of the MEP produced by the TS. The amount of inhibition is expressed as a percentage of the mean MEP amplitude evoked by a single TS. While resting IHI is measured with the subject at rest, active IHI is measured during movement preparation. In healthy subjects executing a hand motor task, the inhibitory effect of one M1 on the other M1 decreases (31) depending on the movement kinematics (32, 33). In a study by Talelli et al. (20), a relationship between resting IHI and task-related ipsilateral M1 activity as measured by fMRI was demonstrated. Specifically, peak forces for a hand grip were positively correlated with increases in ipsilateral M1-blood oxygenation level-dependent (BOLD) response when IHI between motor cortices was weak. This positive correlation changed to a negative correlation when IHI was strong. This would indicate that activity in ipsilateral M1 is controlled to some extent by the inhibitory effect of the contralateral M1.

## Contralesional M1 Reorganization in Post-Stroke Recovery

### Reorganization of contralesional M1 in the post-stroke recovery period (fMRI evidence)

In task-related functional imaging studies of stroke patients, the activation of contralesional motor areas (corresponding to ipsilateral motor areas in healthy subjects) have been consistently reported (34). Cross-sectional studies of stroke patients moving the affected hand revealed a shift from an initially (abnormal) bilateral activation of motor areas in the subacute stroke patients (1, 9, 16, 35–40) toward a more normal unilateral activation pattern of ipsilesional motor areas in chronic stroke patients (40). Importantly, in a longitudinal study of stroke patients, this activation shift to the ipsilesional hemisphere was associated with good recovery, whereas persistence of the bilateral activation pattern was associated with poor outcome (40). On the basis of these studies, it was concluded that greater involvement of contralesional M1 predicted poorer motor outcome. (34, 40). However, in several studies, mirror movements of the non-affected hand were reported during the performance with the affected hand during imaging (34). This raised the possibility that some contralesional M1 activity is, in fact, related to mirror movements of the non-affected hand (41, 42). As mirror movements and coactivation of the non-affected hand are seen more frequently in patients with poor motor outcome (41, 43), the presence of these movements may have confounded the findings of increased contralesional M1 activation in patients with poor outcome.

**Figure 3:**
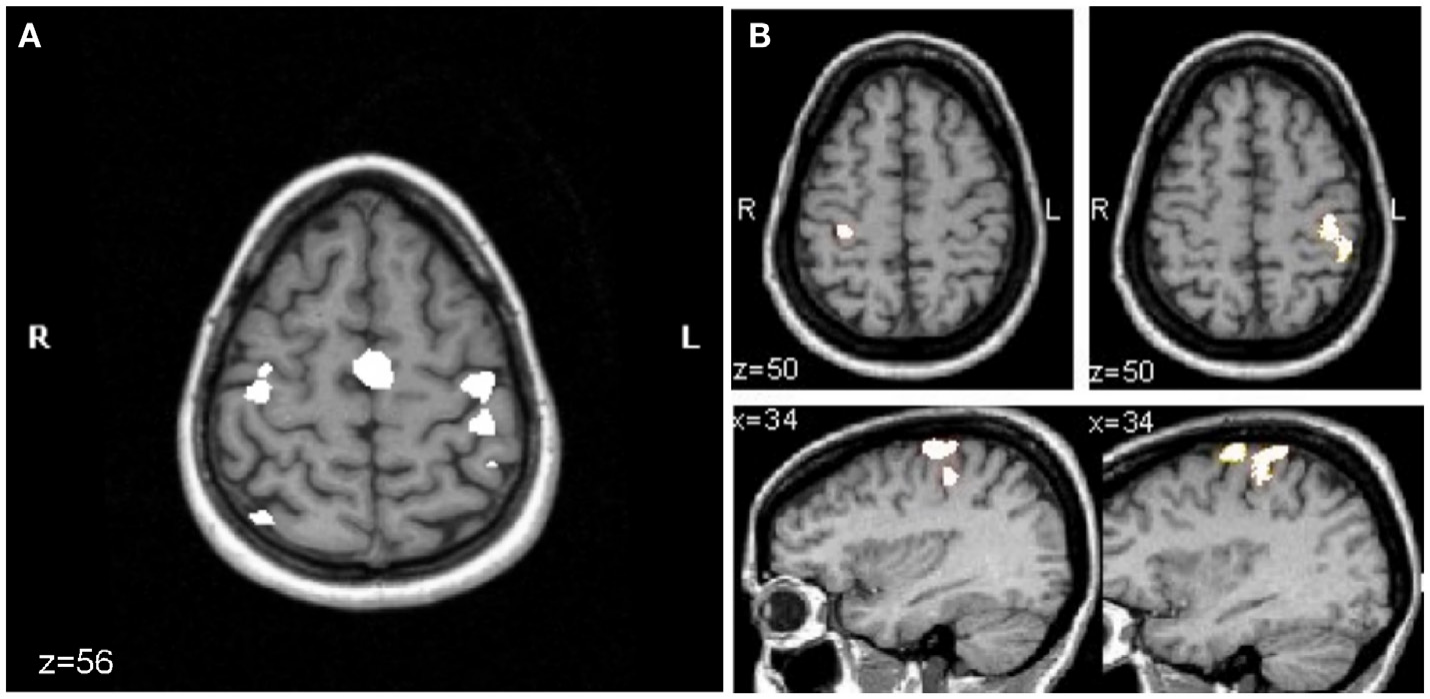
**Mean fMRI activation map of the performance of a finger sequence with the affected hand in patients (*n* = 5) (A) and with either hand in the age-matched control group (*n* = 9) (B)**. For both groups, the activation map is superimposed on the T1-weighted MRI of the same healthy control subject. **(A)** In patients, right in the axial slice of brain (*z* = 56) corresponds to the lesioned hemisphere and left to the contralesional hemisphere. Activation of contralesional precentral gyrus is evident (corrected *p* < 0.05). **(B)** For the control group performing the finger sequence with the left (lower left image, corrected *p* = 0.05) or right (lower right image, uncorrected *p* < 5.8e−12) hand, there was activation in the precentral gyrus of the hemisphere that is contralateral to the performing hand. Initially, the significance level was set as low as corrected *p* = 0.05 to pick up any activity in the motor cortex ipsilateral to the moving hand (shown for left hand movement, lower left image). At this significance level, massive activation was seen in the pre- and postcentral gyrus contralaterally when moving the right hand. To separate clusters of activity in pre- and postcentral gyrus, the significance level was increased until the two clusters became distinct (uncorrected *p* < 5.8e−12, lower right panel) (16).

In our own fMRI study of subacute stroke patients with excellent recovery, strictly unilateral performance resulted in activation of bilateral motor cortices (16). In this study, eight stroke patients underwent fMRI of the brain to test M1 activity related to the performance of a non-sequential finger opposition task with their paretic hand. EMG activity of bilateral arm muscles was recorded during the scanning. All patients showed excellent recovery. Their results were compared to age-matched normal volunteers. While overt mirror movements were absent in all patients, three patients showed substantial EMG activity of the non-affected arm when performing the task with the affected hand. Their data were excluded from further analysis. As demonstrated in Figure 3, in the remaining five patients with strictly unilateral performance, bilateral activation of premotor and primary motor cortices was evident. In contrast, the age-matched controls showed a strictly unilateral activation of the corresponding contralateral M1. These results support the notion that activation in contralesional M1 most likely reflects a reorganizational process in these patients. However, based on the findings in healthy subjects, where ipsilateral M1 is activated as the task becomes more demanding, increased activity could also be explained by a relatively higher demand on motor skill in stroke patients when compared to healthy controls (i.e., because of the compromised hand function due to stroke, the execution of the task is more challenging for the patient compared to the controls). Schaechter and Perdue (44) studied chronic stroke patients with good recovery of hand function and demonstrated that cortical activation during performance of the unskilled and skilled movement was increased in the patients relative to controls in the contralesional primary sensorimotor cortex. These findings suggest that in the chronic phase after stroke the neuronal substrate supporting affected hand function includes contralesional M1. The question whether this abnormal contralesional M1 activity is related to recovery-related regenerative responses as demonstrated for the subacute stroke patients or whether these changes reflect degenerative responses to the stroke remains to be determined as both processes are to some extent activity dependent, interact and impact similar circuitries (4).

### Mechanisms underlying reorganization of contralesional M1 in the post-stroke recovery period

The interpretation of task-related fMRI results is limited by the fact that changes in inhibitory and excitatory activity cannot be distinguished and the functional relevance of these changes in M1 activity is unclear. Specifically, task-related increases in BOLD in contralesional M1 could result from increases of inhibitory or excitatory activity or any combination of these.

In rodent stroke models, functional and structural reorganizational changes in contralesional M1 have been reported [for detailed review, see Ref. (4, 5)]. Briefly, in these models, small focal cortical lesions led to long-lasting changes in contralesional M1, such as down-regulation of GABA_A_-receptor function (45, 46) and up-regulation of NMDA-receptor function (47, 48), both mechanisms operating in increases of synaptic efficacy such as long-term potentiation (LTP). In contrast to human studies (see below), excitability in contralesional M1 was transiently increased but returned to the original values within hours. Similarly, representation of the rodent forelimb expanded in the contralesional M1 but returned to normal dimensions over the following days [for review, see Ref. (5)]. From a structural perspective, increase in neuropil volume (49), use-dependent dendritic growth followed by dendritic pruning, synapse formation, and changes in the specific structure of synaptic connections have been described (49–51).

**Figure 4:**
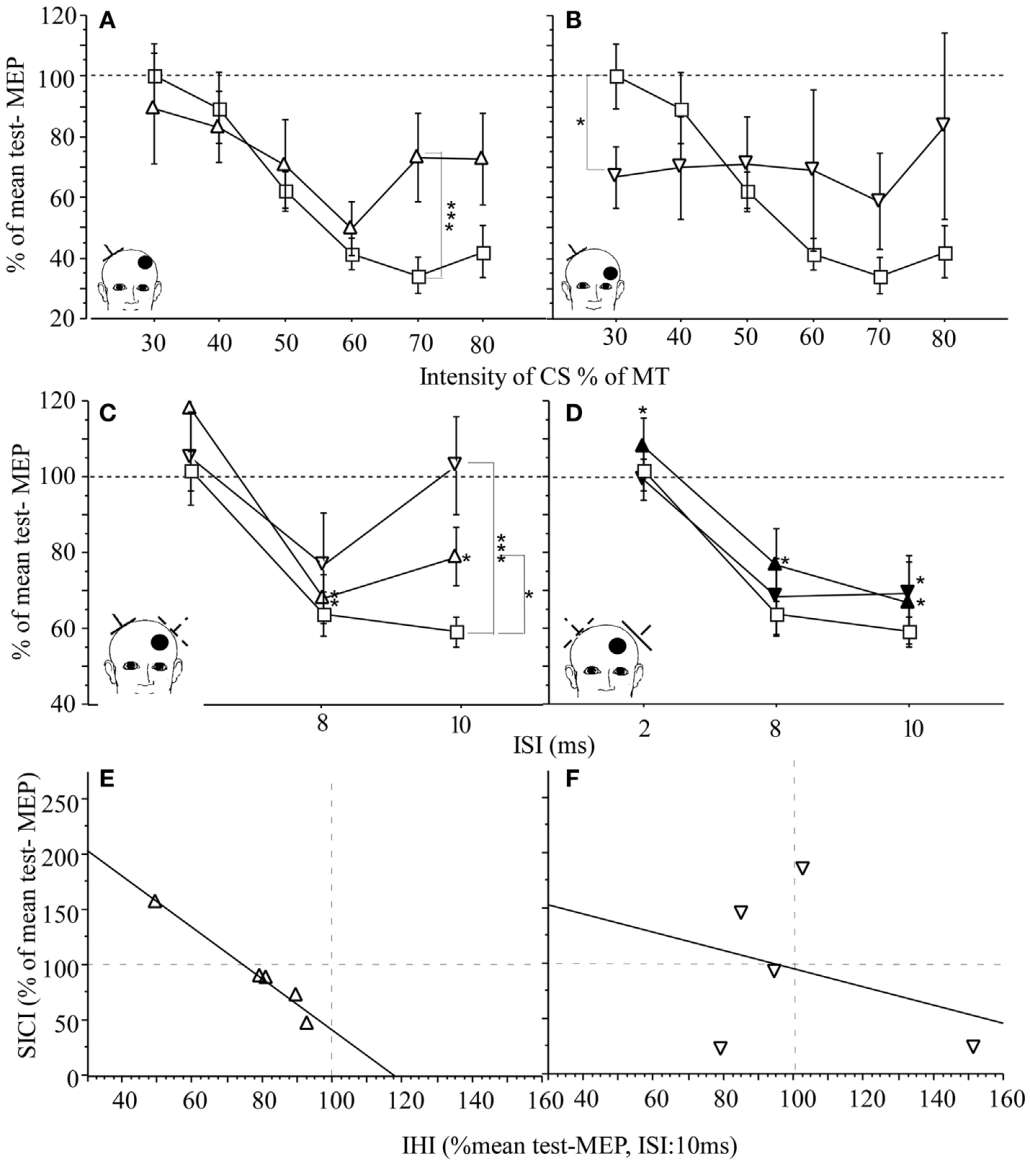
**M1 excitability and IHI in patients with subacute stroke (*n* = 23) and healthy age-matched controls (*n* = 20): EMG was recorded from the first dorsal interosseus muscle (FDI)**. **(A,B)** Effect of lesion location on SICI in patients. Control (square) and contralesional M1 of patients with cortical [open triangle **(A)**] and subcortical location of infarction [open inverted triangle **(C)**]. IHI of the lesioned M1 on the contralesional M1 is reduced in patients with cortical (open triangle) or subcortical infarction (open inverted triangle) when compared to healthy controls (square). **(D)** IHI from contralesional M1 on the lesioned M1 was intact for cortical infarction (black triangle) and subcortical infarction (black inverted triangle). The conditioned MEP amplitude is expressed as percentage of the mean test-MEP. (**E**,**F)** Relationship between M1 excitability, SICI (CS at 80% MT), and IHI in patients with cortical infarction (triangle) and subcortical infarction (inverted triangle). For each patient (each point represents one subject), SICI of the contralesional M1 was plotted against IHI from lesioned on the contralesional M1 (open symbols). Regression was calculated. For cortical location of the infarction, there was an inverse linear relationship between SICE of the contralesional M1 and IHI from lesioned on the contralesional M1 [**(E**) *r*^2^ = 0.972, *p* = 0.002]. Although there is a similar trend in the subcortical group **(F)**, the relationship was more variable [**(F)***r*^2^ = 0.105, *p* = ns]. The insert indicates the position of the coil for application of CS (dotted lines) and the TS (solid lines). The location of the lesion is indicated by the bullet. CS = intensity of conditioning stimulus, MT = motor threshold. The scattered lines indicate the cutoff between facilitation (>100) and inhibition (<100). Mean ± SE. *p < 0.05, **p < 0.02, and ***p < 0.01 (29).

In humans, increased intracortical excitability of contralesional M1 has been demonstrated in subacute and chronic stroke patients (29, 52–54) when explored with the paired pulse TMS technique. In this paradigm, a suprathreshold TS is preceded by a subthreshold CS at an interstimulus interval (ISI) of 2 ms. In the M1 of healthy subjects, CS inhibits the MEP produced by the subsequent TS, referred to as short interval intracortical inhibition (55). This effect is mediated by GABA_A_-receptors (56) and arises in close proximity to the stimulated area (57). By varying the intensity of CS, the effects mediated by inhibitory and excitatory networks can be separated in more detail (29, 54) (Figures 4A,B). In a study of subacute stroke patients, the inhibitory effect of CS at low intensity was similar to values found in healthy age-matched controls while the inhibitory effect was abnormally reduced at higher intensities. This may indicate that the balance of excitatory and inhibitory activity in neuronal circuits was shifted toward excitatory activity (29, 54). Alternatively, abnormal function of the high threshold GABAergic inhibitory interneurons may result in a decreased inhibitory effect of CS at higher intensities. These findings suggest that regulation of excitatory and inhibitory neurotransmitter systems may play a role early in the reorganization process in contralesional M1 (48, 58) and may support functional recovery early after stroke. This notion is supported by the finding in patients in the subacute phase of stroke involving M1 or its corticospinal projections where a close association between increased excitability of contralesional M1 and good recovery of hand function was demonstrated (54). However, whether these findings hold up and can be applied to patients with other lesion locations has to be determined in larger longitudinal studies.

### Relationship between contralesional M1 and ipsilesional M1 (interhemispheric inhibition) in the post-stroke recovery period

As described for the intact brain, the two motor cortices inhibit each other through connections via the corpus callosum (5). In addition to the discussed mechanisms underlying contralesional M1 reorganization, stroke-related changes in the inhibitory drive between motor cortices could play an important role in reorganizational changes of contralesional M1. While increased contralesional M1 excitability was demonstrated in multiple studies (29, 31, 53, 54, 59), very few studies have examined the relationship between increased contralesional M1 excitability and resting IHI. It was concluded that loss of inhibitory drive of the lesioned M1 on the contralesional M1 through interhemispheric connections may contribute to the reorganizational processes observed for this motor cortex. Increases in contralesional M1 excitability may result in an excessive inhibitory effect on the ipsilesional M1, which may interfere with its reorganization and related recovery (31, 53, 59). In our study of 23 subacute stroke patients with documented ongoing recovery of motor function, contralesional M1 excitability was increased as demonstrated by paired pulse TMS technique (29) (see above for detailed description of the methods). Resting IHI from ipsilesional M1 on contralesional M1 was reduced in both cortical and subcortical location of the stroke while IHI from contralesional M1 on ipsilesional M1 was normal (Figures 4C,D). In patients with cortical stroke, there was an inverse correlation between inhibitory effect from contralesional on ipsilesional M1 and contralesional M1 excitability. This relationship was not seen in patients with subcortical stroke. This would indicate that in subacute patients recovering from stroke, the demonstrated increased contralesional M1 excitability is not causally related to abnormally reduced IHI from ipsilesional M1 on contralesional M1. Further, because IHI of the contralesional on ipsilesional M1 was normal and measures of contralesional M1 excitability were increased, there was no evidence in this study to support the hypothesis that an abnormally increased contralesional M1 excitability results in abnormally increased IHI of contralesional on ipsilesional M1 with subsequently decreased activity or excitability of ipsilesional M1 in this patient population. However, when IHI was measured in the pre-movement interval (active IHI, see above for details of the methods) contralesional on the ipsilesional M1 was abnormally increased in chronic stroke patients when compared to healthy age-matched controls (31). The role of abnormally increased active IHI and the relationship between abnormal active IHI, measures of M1 excitability, and recovery of hand function in stroke needs to be determined in more detail and is currently a topic of active investigations.

There is some evidence regarding the relationship between the ipsi- and contralesional M1 in rodent stroke models. Specifically, an ischemic lesion of M1 leads to partial denervation of the contralesional M1, which has a tendency to sprout into the perilesional neuronal tissue of ipsilesional M1 (60, 61). Moreover, learning a new motor skill with the non-affected limb reduces spontaneous recovery and limits rehabilitation-related functional improvements of the affected limb (62–64). These findings underscore the importance of interhemispheric connections between and ipsi- and contralesional M1 and their potential involvement in mediating reorganizational effects on the ipsilesional M1.

### Factors that determine the role of contralesional M1 in the post-stroke recovery period

The factors that determine involvement of contralesional M1 are currently not known. In non-human primate stroke models, progressively larger M1 hand lesions were associated with a proportional expansion of ipsilesional ventral premotor (PMv) (65, 66) and supplementary motor area (SMA) (67) hand representation.

In rodent stroke models, reorganizational changes in contralesional M1 depend on the lesion size (68) and rehabilitation treatment (64, 69) and include long-term changes in neurotransmitter systems, dendritic growth, and synapse formation (45, 46, 50, 51, 70, 71). Inhibiting the contralesional hemisphere in rats that recovered from large ischemic infarcts generates more behavioral deficits of the impaired forelimb in comparison to control animals (72).

In humans, Schaechter and Perdue (44) demonstrated in chronic stroke patients a linear relationship between abnormally increased affected hand movement-related contralesional M1 activity and extend of CST damage. Further, the observed differential effect on contralesional M1 excitability and the relationship between contralesional M1 excitability and IHI (Figure 4) (29) supports the notion that location of the stroke seems to impact reorganizational processes. These differential remote effects of the lesion are also consistent with the findings that contralesional M1 seems to support function in a subset of patients after stroke (18) but may interfere with recovery or affected hand function in others (73, 74).

## Interventions in Stroke Rehabilitation Treatment Targeting Contralesional M1

Several reports have demonstrated that non-invasive cortical stimulation can enhance functional reorganization, motor cortical excitability, and the beneficial effects of motor training on performance (75–80). Either ipsi- or contralesional M1 are target of these interventional approaches (3). In this review, I will focus on non-invasive cortical stimulation targeting the contralesional M1.

Down-regulation of excitability in one motor cortex influences corticomotor excitability in the opposite motor cortex. Several reports of studies in healthy subjects have now demonstrated that 1 Hz rTMS applied to M1 of one hemisphere results in increased corticomotor excitability in the opposite M1 (81, 82) and improved performance in the corresponding hand (14, 83) depending on the level of motor demand (14). As discussed in the previous sections, although the extent to which the contralesional M1 contributes to motor recovery is not known, many currently employed rTMS protocols are designed with the assumption that following stroke, ipsilesional M1 is hypoactive while contralesional M1 is hyperactive and should be inhibited (3, 80). Accordingly, stimulation of contralesional M1 has been used to inhibit its hyperactivity (3, 74, 78, 84–86). Meta- analyses on the effectiveness of repetitive transcranial magnetic stimulation (rTMS) or transcranial direct current stimulation (tDCS) in stroke rehabilitation therapy do not agree on the available evidence to either support or reject it (87–90).

## Summary

Taken together, there is evidence from human and animal studies that activity in contralesional M1 will impact motor function of the paretic limb differently in different patients. However, currently employed treatment strategies are geared toward inhibiting its function. There is a great need to identify the precise factors that specifically influence the role of contralesional M1 in the recovery process. A better understanding of those factors is critical to the development of effective therapies tailored to its specific role in the recovery process to improve outcome post stroke.

## Conflict of Interest Statement

The author declares that the research was conducted in the absence of any commercial or financial relationships that could be construed as a potential conflict of interest.
